# Neural Correlates of Motor/Tactile Imagery and Tactile Sensation in a BCI paradigm: A High-Density EEG Source Imaging Study

**DOI:** 10.34133/cbsystems.0118

**Published:** 2024-06-21

**Authors:** Huan Wen, Yucun Zhong, Lin Yao, Yueming Wang

**Affiliations:** ^1^ The Department of Neurobiology, Affiliated Mental Health Center & Hangzhou Seventh People’s Hospital, Zhejiang University School of Medicine, Hangzhou, China.; ^2^ The Nanhu Brain-Computer Interface Institute, Hangzhou, China.; ^3^ The MOE Frontiers Science Center for Brain and Brain-Machine Integration, Zhejiang University, Hangzhou, China.; ^4^ The College of Computer Science, Zhejiang University, Hangzhou, China.; ^5^ The College of Biomedical Engineering & Instrument Science, Zhejiang University, Hangzhou, China.; ^6^ The Qiushi Academy for Advanced Studies, Hangzhou, China.

## Abstract

Complementary to brain–computer interface (BCI) based on motor imagery (MI) task, sensory imagery (SI) task provides a way for BCI construction using brain activity from somatosensory cortex. The underlying neurophysiological correlation between SI and MI was unclear and difficult to measure through behavior recording. In this study, we investigated the underlying neurodynamic of motor/tactile imagery and tactile sensation tasks through a high-density electroencephalogram (EEG) recording, and EEG source imaging was used to systematically explore the cortical activation differences and correlations between the tasks. In the experiment, participants were instructed to perform the left and right hand tasks in MI paradigm, sensory stimulation (SS) paradigm and SI paradigm. The statistical results demonstrated that the imagined MI and SI tasks differed from each other within ipsilateral sensorimotor scouts, frontal and right temporal areas in α bands, whereas real SS and imagined SI showed a similar activation pattern. The similarity between SS and SI may provide a way to train the BCI system, while the difference between MI and SI may provide a way to integrate the discriminative information between them to enhance BCI performance. The combination of the tasks and its underlying neurodynamic would provide a new approach for BCI designation for a wider application. BCI studies concentrate on the hybrid decoding method combining MI or SI with SS, but the underlining neurophysiological correlates between them were unclear. MI and SI differed from each other within the ipsilateral sensorimotor cortex in alpha bands. This is a first study to investigate the neurophysiological relationship between MI and SI through an EEG source imaging approach from high-density EEG recording.

## Introduction

Mental imagery is the overt basis of human cognitive abilities [1]. Motor imagery (MI) is one of the most common imagery cognitive processes, in which subjects only need to perform the imagination of motor action (e.g., left- or right-hand movement) without any execution [2]. MI decoding in brain–computer interface (BCI) systems has been successfully applied in robot control [3], game playing [4], rehabilitations for strokes [5], etc. Distinguished from other BCI systems such as steady-state visual evoked potentials [6] and P300 [7], MI-based BCIs can voluntarily modulate frequency-specific sensorimotor rhythms mainly extracted from the sensorimotor cortex and do not require any external stimuli [8].

In comparison to the MI task, sensory imagery (SI) also called tactile imagery is another type of mentally cognitive process in the somatosensory system. A series of studies have shown that the intention of tactile/haptic sensation can be reliably decoded by brain signals such as steady-state somatosensory evoked potentials [9] and tactile event-related desynchronization/synchronization (ERD/ERS) [10–12]. Moreover, we have shown that subjects can learn to perform the imagined sensation tasks, even when no real tactile stimulus was applied. This imagined tactile task is feasible for BCI construction, which has benefits of increasing BCI diversities within a stimulus-independent BCI framework [13–17].

Both mental imagery tasks (MI and SI) and sensory stimulation (SS) task induce rhythmic changes in the contralateral hemisphere, which can be quantified by ERD from different frequency bands [10]. By combining different mental tasks and underlining the ERD activity, it has the potential to improve the classification accuracy of the BCI system and increase the limited number of output commands. Because of the similar activation pattern among the 3 tasks, research [18] has investigated the relationship between SI and SS by calculating mu and beta ERD/ERS on electrode levels. The results show that SI differs from SS mainly in the contralateral hemisphere (left sensorimotor areas). Currently, the quantification of task-related electroencephalogram (EEG) analysis (MI and SI) is mainly focused on decoding tasks and the electrode level, which has limited power to distinguish the underlying neurodynamic on the cortex level.

Complementary to those EEG studies, the relationship between SS-induced and SI-induced brain oscillatory has been investigated by functional magnetic resonance imaging (fMRI), which provides a higher spatial resolution. It has been shown that neural substrates of primary sensory area (SI), secondary sensory area (SII), and superior parietal lobe area are activated during tactile imagery [19], and SS paradigms share similar neural representations [14,20]. Recent neuroimaging works suggest that Brodmann area 3a (BA3a) and partial BA2 that overlay the postcentral gyrus can deal with proprioceptive signals, while BA3b and BA1 are mainly dominated by cutaneous receptors [21,22]. Moreover, fMRI studies have also pointed out that primary motor cortex (Brodmann’s area 4), premotor area (Brodmann’s area 6), parietal areas, and supplementary motor area (Brodmann’s area 6) were activated during the MI task [23–25]. Another fMRI study [26] states that MI is mainly dependent on central processing mechanisms, which play a role in generating and planning complex motor movements. Plenty of neuroimaging works have demonstrated the sensory/motor recruitment during tactile/MI in 2 disparate fields as mentioned above. However, no direct research to date has studied the neural correlates between SI and MI oscillatory dynamics, especially on the cortex level. Besides, fMRI imaging modality’s lack of temporal resolution makes it difficult to investigate the underlying neurodynamic correlations between the real and imagined tasks, which are important for BCI construction for real-time control.

In this study, to identify which scout revealed differences between MI and SI, we focused on exploring the differences between 2 mental imagery tasks and the real stimulation task by EEG source imaging (ESI) technique. This technique is usually used to counterweigh the volume conduction effect from EEG by projecting scalp signals to the cortex of the brain. ESI technique has already been utilized to compute ERD/ERSs in MI and can improve the performance of classification tasks [27,28]. Furthermore, our previous study [29] applied the ESI technique to high-density EEG data, demonstrating a similar discriminative information distribution between SI and SS task on the cortex level. Therefore, a high-density EEG signal with ESI would provide a tool to recover the neurophysiological origins during the imagined SI and MI tasks. In this study, results from the underlying neurodynamic of motor/tactile imagery and tactile sensation tasks can be utilized to enhance BCI performance in many applications such as MI and SI hybrid decoding or real-time BCI control.

## Materials and Methods

### Subjects

Eleven healthy BCI naïve subjects participated in the experiments (6 female and 5 male; average age, 23.5 ± 1.5 years). Our recruitment announcement for participants was posted on the official forum of Zhejiang University. The recruitment criteria are as follows: First, participants must be naïve to our paradigm. Second, they must be right-handed. In addition, they must self-report being under good physical condition with normal or corrected-to-normal vision. All participants signed informed consent forms before participation.

### Tactile stimulation

Two linear resonant actuators (10 mm; C10-100, Precision Microdrives Ltd.) were utilized to administer tactile stimulation, typically with a normalized amplitude of 1.4*g*, applied to the dorsal side of the wrists. The device controlled by a soundcard could generate a 27-Hz sine wave modulated with a 175-Hz sine carrier wave. This stimulation aimed to activate both Pacinian corpuscles, sensitive to frequencies above 100 Hz, and Meissner corpuscles, sensitive to frequencies ranging from 20 to 50 Hz [30]. The amplitude of the stimuli could be adjusted individually within a range from 0.5 times the device’s normalized amplitude to a maximum amplitude of 11.3 μm at the resonant frequency.

### Experimental paradigm

**Fig. 1.**
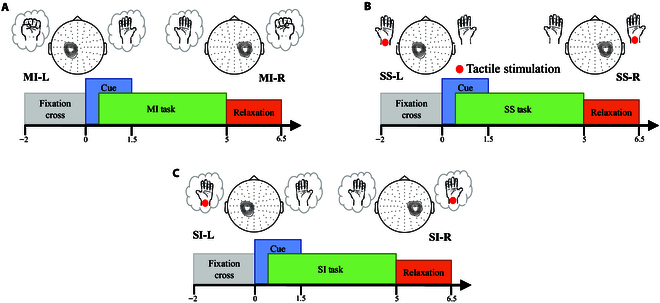
Graphic illustration of 3 experimental paradigms. (A) MI paradigm. (B) SS paradigm. (C) SI paradigm.

In our experiment, the entire session comprised 3 sequential blocks, each completed by the subjects in chronological order. Each block comprised a total of 80 trials with 40 trials per run. Trials for different hands were randomly present during the experiment. Resting periods of 5 to 10 min between blocks were provided to avoid the fatigue of subjects. In the first block, the subjects were required to perform left- or right-hand MI. In the second block, subjects were instructed to focus on the left or right wrist that received the real tactile stimuli (SS). In the third block, subjects were instructed to imagine sensations on the left or right hand when there were no tactile stimuli (SI task). The specific illustration of the 3 blocks is shown in Fig. 1.

#### MI paradigm

The experimental paradigm is illustrated in Fig. 1A. At the beginning of each trial (*T* = −2 s), a white fixation cross (“+”) appeared in the center of the screen. At *T* = 0 s, a red visual cue that lasted for 1.5 s appeared randomly either left or right on the computer monitor: (a) a left-pointing cue corresponding to left MI (MI-L) and (b) a right-pointing cue corresponding to right MI (MI-R). The subjects were required to continue imagining the motor movement until the disappearance of the fixation cross. At *T* = 5 s, a few seconds of relaxation was followed before the next trial began. Each subject performed 2 runs in this block with 20 left and 20 right per run.

#### SS paradigm

The experimental procedure was the same as described above, whereas subjects were instructed to sense the real tactile stimuli, as shown in Fig. 1B. At *T* = 0 s, a red visual cue was randomly presented either left or right on the computer monitor. Subjects were instructed to focus on the left (SS-L) or right (SS-R) wrist where the tactile stimuli were applied. The stimuli continued for 5 s until the white fixation cross disappeared.

#### SI paradigm

The experimental paradigm is illustrated in Fig. 1C. In this block, subjects were required to shift and maintain the somatosensory attention on the left or right hand without any tactile stimuli. The experiment protocol was the same as MI and SS tasks: (a) a left-pointing cue referring to left SI task (SI-L) and (b) a right-pointing cue referring to right SI task (SI-R).

### EEG recording and preprocessing

The EEG signal was captured using the BioSemi ActiveTwo EEG amplifier (BioSemi Inc., Amsterdam, the Netherlands) with a high-density (128-channel) active head cap. The electrodes were positioned based on the BioSemi ABC position system. The common mode sense active electrode served as the reference, while the driven right leg passive electrode served as the ground. In addition, 2 active electrodes were positioned at the left and right mastoids for re-referencing. Throughout the recording session, the offset magnitude of all electrodes to the common mode sense electrode was maintained within ±25 mV, and the signals were sampled at a rate of 512 Hz. High-density EEG signals were recorded in a row during MI, SI, and SS. Each paradigm contained 80-trial data with 240 trials in total. A standard EEG preprocessing pipeline was applied using EEGLAB toolbox [31]. First, a sixth-order Butterworth notch filter (49 to 51 Hz) was adopted to remove power-line noise. Then, all data were restricted to the alpha–gamma band (7 to 50 Hz) by a sixth-order Butterworth band-pass filter. A common average reference was applied. Second, independent component analysis was used to correct artifacts, such as eye blinks and muscle movement. After that, trials exceeding ±100 μV were removed.

### EEG source imaging

In this paper, Brainstorm toolbox [32] was used to perform ESI. Before solving the electromagnetic inverse problem, the lead field matrix was calculated by the boundary element method model [33] that was constructed from default DKomical MRI and ICBM152 template brain [34]. The standardized low-resolution electromagnetic tomography (sLORETA) method [35] was used to obtain 15,002-dipole cortical activities. The formula of sLORETA method is as follows [35]:

$$\phi =\mathrm{LJ}+c_{1}$$ (1)

where *ϕ* ∈ *R*^*N_E_*×*t*^ represents the preprocessed EEG signals with *N_E_* electrodes and *t* time points. *L* ∈ *R*^*N_E_*×*N_D_*^ is the lead field matrix, and *N_D_* is the number of dipoles. *J* ∈ *R*^*N_D_*×*t*^ reveals the signals with *t* time points of all *N_D_* dipoles. *c*_1_ refers to the noise of signals.

This highly underdetermined Eq. 1 can be solved by standardization of the minimum norm inverse solution whose equation of interest is as follows:

$$\underset{\text{min}}{F}={\left\|\phi -\mathrm{LJ}-c_{1}\right\|}^{2}+\lambda {\left\|J\right\|}^{2}$$ (2)

where *λ* refers to the regularization parameter that is always greater than zero.

Given the EEG signal *ϕ* and lead field *L*, the final solution to this minimization problem is:

$$\hat{J}=L^{T}H{\left[{\mathrm{HLL}}^{T}H+\lambda H\right]}^{+}\phi$$ (3)

where *H* reveals the centering matrix and the sign []^+^ is Moore–Penrose pseudo-inverse of a matrix.

Since the number of dipoles was large and the electrical activity of the neighbor dipoles can be highly correlated, the Desikan–Killiany atlas [36] in the toolbox was adopted, and all dipoles were gathered into different independent scouts, also named ROIs (regions of interest). The following analysis in the source domain was performed on the basis of those nonoverlapping scouts. Figure S1 represents the brain map of the Desikan–Killiany atlas in 8 different views. Furthermore, Table S1 lists names of each independent atlas area corresponding to labels shown in Fig. S1.

### ERD/ERS calculation

In general, frequencies of brain signals are correlated with their amplitude. Event-related desynchronization and event-related synchronization could just display frequency-specific relative values, e.g., percentages, of the decreased or increased power with the baseline period (usually defined as a few seconds before a motor or sensory event) [37]. To measure the values of ERD/ERS, preprocessed EEG signals or the signals in the source domain were filtered into 7 frequency bands: [8 10] Hz (α1), [10 13] Hz (α2), [13 20] Hz (β1), [20 26] Hz (β2), [26 30] Hz (β3), [30 35] Hz (γ1), and [35 40] Hz (γ2). Hilbert transform [38] was then used to obtain the envelopes of amplitudes *A*(*t*), based on filtered signals from different frequency bands. After that, ERD/ERS was calculated according to signal power of various rhythms. The ERD/ERS can be defined as followed [37]:

$$\mathrm{ERD}/\mathrm{ERS}=\frac{{A_{T}}^{2}-{A_{B}}^{2}}{{A_{B}}^{2}}\times 100\%$$ (4)

where *A_T_* refers to the envelopes of amplitudes during the motor or sensory event and *A_B_* refers to the averaged envelopes from the baseline period.

### Statistical analysis

Statistical analysis was conducted using MATLAB R2020b. The baseline period and time of interest were from −1.5 to −1 s and 1.5 to 4.5 s, respectively. As suggested by previous research [37,39,40], averaged samples from long segment did not offer enough statistical information, so in many applications [37,39,41], EEG signals were split into smaller pieces to get constant stationary features from multivariate time series. Besides, the objective of the present research is to investigate which scouts are statistically different between groups per subfrequency band. Therefore, the split-time courses of ERD/ERS were averaged to reduce variabilities and increase the sample size. Following that, a one-way test was used. Trial-averaged ERD/ERS values were divided by a nonoverlapped sliding time window with a 0.25-s interval and averaged. That is, each neighbor/scout consisted of 12 samples per subject.

A cluster-based permutation test with 5,000 times was used to assess differences between each block of data [42]. As mentioned above, dipoles were divided into 500 independent neighbors for each subgroup (SI/SS/MI). Data of the left and right hand were analyzed respectively across all subjects (*N* = 11). The *P* value for maximum statistics and test-wise *P* value for cluster inclusion were set to 0.05 and 0.001, respectively. A 2-tail comparison was used afterward.

## Results

### ERD/ERS in 3 tasks

**Fig. 2.**
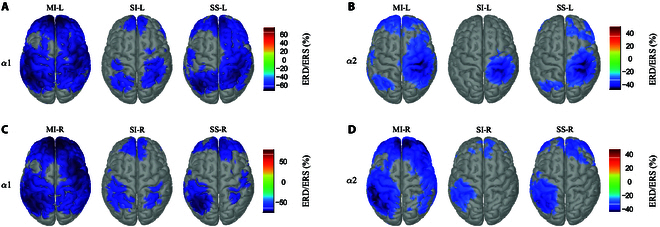
Brain map of grand-averaged ERD/ERS within α bands. (A) Left hand (LH) ERD/ERS activation in α1. (B) LH ERD/ERS activation in α2. (C) Right hand (RH) ERD/ERS activation in α1. (D) RH ERD/ERS activation in α2.

Figure 2 shows the grand-averaged ERD/ERS distribution in source domains for left and right MI/SI/SS tasks, respectively. Only α frequency bands were illustrated according to statistical results. ERD/ERS distribution for β rhythms (Fig. S2) was illustrated in the Supplementary Materials, which showed almost no statistical difference in MI versus SI comparison.

For the left hand (LH) task in α1 (Fig. 2A), both imagery task (SI-L) and stimulation task (SS-L) revealed a right contralateral activation (ERD), whereas MI-L showed a more distinctive bilateral activation as compared with others. Within α2 rhythm (Fig. 2B), the contralateral right hemisphere activation (LH ERD) occurred in all 3 tasks. The threshold settled for the ERD/ERS representation in Fig. 2A and B was 74% and 70% of the maximum value, respectively.

During the right hand (RH) task, ERDs/ERSs in the source domain revealed similar cortical activities. As for α1 band (Fig. 2C), all 3 tasks illustrated a distinctive left contralateral activation (ERD) with MI-R showing a wider ERD/ERS distribution in both hemispheres. Nevertheless, ERD/ERS components in α2 rhythm (Fig. 2D) revealed a contralateral left desynchronization in all 3 tasks. The settled threshold for α1 and α2 bands was 86% and 74% of the corresponding maximum, respectively.

The frontal cortex also was activated during MI/SS task no matter the frequency bands and hand sides. Moreover, magnitudes of ERD/ERS in both hands exhibited a reduced distribution from α1 to α2 rhythm.

### Statistical analysis in source domain

#### Task differences in alpha rhythm

**Fig. 3.**
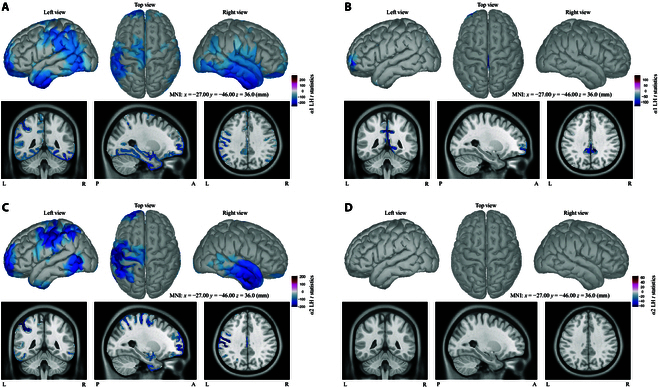
Brain map of *H* statistics within α bands for LH task in the form of 3 views (extern left, top, and extern right) and MRI format. (A and C) Cluster-based *t* statistics of MI-L versus SI-L group with respect to α1 andα2 rhythms. (B and D) *t* statistics of SI-L versus SS-L group with respect to α1 and α2 rhythms.

**Fig. 4.**
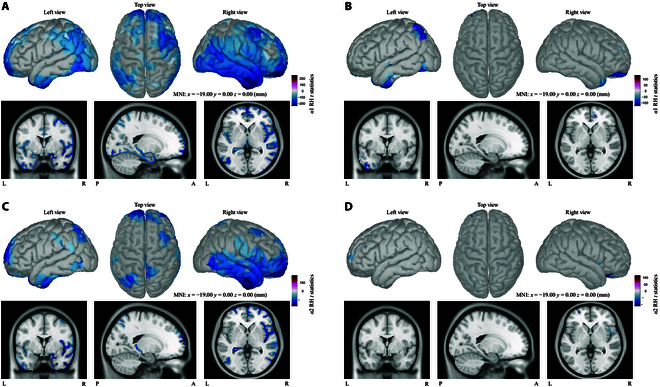
Brain map of H statistics within α bands for RH task in the form of 3 views (extern left, top, and extern right) and MRI format. (A and C) Cluster-based *t* statistics of MI-R versus SI-R group with respect to α1 and α2 rhythms. (B and D) *t* statistics of SI-R versus SS-R group with respect to α1 and α2 rhythms.

Figures 3 and 4 illustrate brain maps of *t* statistics between SI versus MI/SS comparisons in LH and RH, respectively. Only statistically significant scouts (*P* ≤ 0.05) were represented by different colors within 2 frequency bands (α1 and α2). Partial dipoles were divided into multiple independent neighbors to better distinguish which scout/cluster is statistically different on the basis of the Desikan–Killiany atlas as revealed in Fig. S1 and Table S1. The number of independent neighbors was settled as 500 for the cost of computation, and the effect of the number (from 500 to 1,500) of ROIs on statistical results will be further discussed.

Pronounced significances between SI-L and MI-L were found in the α1–α2 band (Fig. 3A and C), whereas SI and SS showed almost no topographical differences (Fig. 3B and D). Concretely in Table 1, the significantly different areas between SI and MI overlayed fractional regions of rostral middle frontal, superior frontal, precentral, postcentral, and paracentral scouts for α1 (Fig. 3A) and α2 (Fig. 3C). Moreover, within the right inferior, middle temporal, and insula cortex, statistical differences occurred in both alpha bands.

**Table 1.** Clusters with maximum *t* statistics in MI-L versus SI-L comparison

Fre	Hem	DK	*X*	*Y*	*Z*	Max*T*
α1	R	Fusiform	35 ± 5	−21 ± 5	−33 ± 5	−196.12
	R	Inferior temporal	48 ± 8	−2 ± 6	−44 ± 5	−205.39
	R	Insula	42 ± 2	−4 ± 8	−7 ± 8	−182.28
	L	Lateral occipital	−47 ± 4	−72 ± 4	−5 ± 6	−179.91
	R	Middle temporal	47 ± 7	6 ± 4	−41 ± 4	−210.42
	L	Postcentral	−55 ± 4	−24 ± 5	52 ± 7	−175.58
	L	Rostral middle frontal	−31 ± 7	61 ± 4	−4 ± 5	−178.2
	L	Supramarginal	−62 ± 5	−39 ± 2	29 ± 4	−177.01
α2	L	Inferior temporal	−56 ± 7	−64 ± 4	−10 ± 6	−173.28
	R	Insula	42 ± 2	−4 ± 8	−7 ± 8	−144.16
	L	Lateral occipital	−47 ± 4	−72 ± 4	−5 ± 6	−163.65
	R	Middle temporal	56 ± 6	5 ± 5	−23 ± 5	−185.31
	L	Paracentral	−3 ± 2	−27 ± 4	57 ± 7	−137.68
	L	Postcentral	−54 ± 4	−23 ± 5	53 ± 8	−186.66
	L	Posterior cingulate	−5 ± 9	−18 ± 7	41 ± 5	−154.97
	L	Precentral	−54 ± 5	−9 ± 4	47 ± 8	−178.51
	L	Rostral middle frontal	−27 ± 3	64 ± 4	9 ± 9	−166.75
	L	Superior frontal	−18 ± 4	68 ± 4	12 ± 4	−148.15
	L	Superior parietal	−42 ± 4	−46 ± 4	52 ± 9	−177.97
	R	Superior temporal	51 ± 5	13 ± 6	−25 ± 6	−161.77
	L	Supramarginal	−49 ± 5	−40 ± 10	50 ± 5	−187.74

Fre, frequency; Hem: hemisphere; DK, Desikan–Killiany altas; *XYZ*, clustered mean Montreal Neurological Institute (MNI) coordinates with SD; max*T*, maximum *t* value.

As for RH case, statistical differences between SI and MI were mostly found in rostral middle frontal, superior frontal, caudal middle frontal, right precentral, right paracentral, right temporal, and left superior parietal areas (Fig. 4A and C). Comparisons between SI and SS for both α rhythms (Fig. 4B and D) shared almost no statistically different regions, which was similar to the LH comparison. The concrete statistical results were listed in Table 2 with cluster-based max *t* values. Both Tables 1 and 2 showed the top 10% *t* values from statistical analysis of MI versus SI group.

**Table 2.** Clusters with maximum *t* statistics in MI-R versus SI-R comparison

Fre	Hem	DK	*X*	*Y*	*Z*	Max*T*
α1	L	Caudal anterior cingulate	−7 ± 4	20 ± 6	31 ± 5	−158.03
	R	Caudal middle frontal	40 ± 7	17 ± 8	57 ± 5	−175.06
	R	Fusiform	43 ± 6	−64 ± 5	−15 ± 3	−184.41
	R	Inferior parietal	48 ± 7	−60 ± 2	23 ± 5	−177.13
	R	Inferior temporal	48 ± 8	−2 ± 6	−44 ± 5	−217.05
	R	Insula	42 ± 2	−4 ± 8	−7 ± 8	−187.18
	R	Lateral occipital	49 ± 4	−73 ± 4	−6 ± 3	−179.57
	R	Lateral orbitofrontal	29 ± 4	37 ± 3	−15 ± 4	−161.25
	L	Lingual	−10 ± 6	−89 ± 5	−15 ± 3	−150.13
	R	Medial orbitofrontal	5 ± 3	49 ± 5	−12 ± 4	−164.82
	R	Middle temporal	47 ± 7	6 ± 4	−41 ± 4	−226.46
	R	Precentral	32 ± 5	−7 ± 4	56 ± 6	−163.38
	R	Rostral middle frontal	30 ± 5	55 ± 4	13 ± 4	−188.74
	L	Superior frontal	−7 ± 4	60 ± 6	4 ± 4	−183.46
	L	Superior parietal	−30 ± 3	−53 ± 3	47 ± 5	−168.42
	R	Superior temporal	51 ± 5	13 ± 6	−25 ± 6	−201.44
α2	R	Caudal middle frontal	39 ± 6	18 ± 7	57 ± 4	−109.09
	L	Fusiform	−43 ± 2	−22 ± 7	−26 ± 7	−122.58
	L	Inferior parietal	−33 ± 5	−80 ± 4	43 ± 5	−109.9
	R	inferior temporal	48 ± 8	−2 ± 6	−44 ± 5	−161.69
	R	Insula	42 ± 2	−4 ± 8	−7 ± 8	−149.64
	L	Isthmus cingulate	−5 ± 4	−43 ± 5	32 ± 5	−136.43
	R	Lateral occipital	49 ± 4	−73 ± 4	−6 ± 3	−144.33
	R	Lateral orbitofrontal	22 ± 3	30 ± 4	−21 ± 4	−111.35
	R	Middle temporal	47 ± 7	6 ± 4	−41 ± 4	−151.7
	R	Paracentral	6 ± 5	−39 ± 4	60 ± 7	−108.88
	R	Parstriangularis	54 ± 4	27 ± 4	0 ± 9	−119.63
	R	Posterior cingulate	7 ± 5	−22 ± 5	42 ± 5	−137.44
	R	Precentral	60 ± 4	7 ± 5	10 ± 7	−111.52
	L	Precuneus	−5 ± 4	−43 ± 4	43 ± 5	−137.41
	R	Rostral middle frontal	30 ± 5	55 ± 4	13 ± 4	−160.68
	L	Superior frontal	−17 ± 5	68 ± 4	12 ± 4	−153.25
	L	Superior parietal	−29 ± 5	−66 ± 5	58 ± 6	−136.96
	R	Superior temporal	51 ± 5	13 ± 6	−25 ± 6	−129.8

In general, the statistical results revealed a task-related difference between MI and SI, with left sensorimotor regions for the left hand task (MI-L versus SI-L), while right sensorimotor regions for the right hand task (MI-R versus SI-R). Besides, partial frontal and right temporal areas along with superior parietal regions also illustrated distinctive differences no matter in left- or right-hand task.

#### Task differences in gamma rhythm

**Fig. 5.**
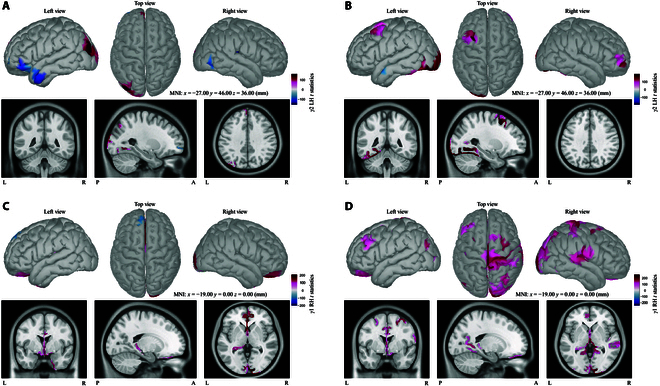
Brain map of cluster-based *t* statistics within γ2 for LH and γ1 for RH in the form of 3 views (extern left, top, and extern right) and MRI format. (A) *t* statistics in γ2 of MI-L versus SI-L comparison. (B) Statistical results of SI-L versus SS-L in γ2. (C and D) *t* statistics in γ1 rhythm of MI-R versus SI-R and SI-R versus SS-R groups, separately.

Interestingly, there were also differences between SI and SS in γ2 rhythm for LH (Fig. 5B) and γ1 rhythm for RH (Fig. 5D), respectively. Compared to MI versus SI group, statistically pronounce regions in SI-L versus SS-L comparison were found mostly in left superior frontal and lateral occipital areas. Unlike LH task, RH (SI-R versus SS-R) occupied larger significant areas that mainly overlayed on the sensorimotor cortex such as right precentral, postcentral, and lateral occipital areas. However, opposite to the comparison in alpha bands, MI versus SI group showed almost no differences (Fig. 5A and C). Table 3 represented detailed clusters and statistical results for SI versus SS comparison that only illustrated the top 10% *t* statistics of significant clusters.

**Table 3.** Clusters with maximum *t* statistics in SI versus SS comparison

Fre	Task	Hem	DK	*X*	*Y*	*Z*	Max*T*
γ2	LH	L	Fusiform	−26 ± 3	−63 ± 7	−11 ± 3	68.86
		L	Inferior parietal	−38 ± 6	−89 ± 3	21 ± 6	157.67
		R	Inferior temporal	56 ± 3	−67 ± 1	−6 ± 5	−77.55
		L	Insula	−41 ± 2	−2 ± 5	−12 ± 5	−50.68
		L	Lateral occipital	−30 ± 5	−97 ± 2	14 ± 7	111.22
		R	Lateral occipital	50 ± 4	−74 ± 3	−7 ± 4	−106.59
		L	Lingual	−22 ± 5	−66 ± 6	−10 ± 3	106.72
		L	Medial orbitofrontal	−4 ± 2	56 ± 5	−25 ± 4	−50.47
		L	Middle temporal	−53 ± 5	7 ± 6	−36 ± 6	−131.94
		L	Parahippocampal	−21 ± 4	−32 ± 5	−19 ± 5	80.33
		L	Postcentral	−52 ± 3	−24 ± 5	58 ± 5	−55.46
		R	Precuneus	14 ± 2	−65 ± 2	34 ± 2	50.59
		L	Rostral middle frontal	−30 ± 4	61 ± 4	−4 ± 3	−88.1
		L	Superior frontal	−4 ± 2	56 ± 5	29 ± 5	103.34
		L	Superior temporal	−49 ± 4	10 ± 4	−29 ± 5	−109.86
		R	transverse temporal	43 ± 4	−20 ± 3	9 ± 5	−61.37
γ1	RH	R	Caudal anterior cingulate	2 ± 1	21 ± 6	22 ± 4	62.36
		L	Inferior parietal	−41 ± 3	−78 ± 3	35 ± 3	64.92
		R	Isthmus cingulate	6 ± 3	−42 ± 3	7 ± 3	66.7
		R	Lateral occipital	16 ± 6	−103 ± 2	0 ± 7	144.23
		R	Lateral orbitofrontal	11 ± 2	35 ± 9	−24 ± 5	150.44
		L	Lingual	−7 ± 3	−89 ± 5	−15 ± 3	96.01
		L	Medial orbitofrontal	−7 ± 4	24 ± 7	−23 ± 4	171.74
		L	Parahippocampal	−24 ± 5	−28 ± 4	−22 ± 5	78.1
		R	Pericalcarine	10 ± 4	−85 ± 3	7 ± 2	110.85
		R	Postcentral	13 ± 5	−31 ± 3	77 ± 4	72.52
		R	Posterior cingulate	8 ± 5	−21 ± 4	42 ± 3	97.36
		R	Precuneus	21 ± 3	−59 ± 2	22 ± 5	62.14
		R	Rostral anterior cingulate	2 ± 3	39 ± 5	9 ± 8	167.25
		L	Rostral middle frontal	−49 ± 4	33 ± 4	31 ± 6	66.61
		R	Superior frontal	6 ± 4	52 ± 3	10 ± 4	177.52

Task, type of the task (left or right hand).

## Discussion

In this work, neural correlates of motor/tactile imagery and tactile stimulation were systematically investigated through a high-density EEG study with the source imaging approach. According to previous investigations [10,41,43], EEG oscillation in both imagery and real stimulation tasks revealed frequency-specific power decrease/increase in the contralateral/ipsilateral hemisphere (ERD/ERS). On the basis of this discriminative information, MI-, SI-, and SS-based BCIs have been utilized to decode human motor/sensory intentions, and various research demonstrated that a combination of them can improve the performance of BCIs. The hybrid modality BCI combined brain signals from the motor, and somatosensory cortex (MI and SI) indicated that SI-L/MI-R or SI-R/MI-L could result in higher classification accuracy where distinguish differences in alpha-band ERD/ERS activation were observed between SI ERD/ERS (LH task) and MI ERD/ERS (RH task) [44]. Another combination modality was achieved by using real stimulations to enhance the ERD/ERS activation in MI. Discrimination between MI-L and MI-R was improved by the tactile stimulation assistance that could be concretely embodied as a stronger time continuity in C3/C4 electrode [15,45]. Plenty of research concentrated on obtaining a better performance on hybrid sensorimotor rhythm BCIs, but few [14,18,19] aimed to investigate the relationship among them especially on cortical level. From this point of view, in this study, we have verified that ERD/ERS values from SI and MI in both hand cases differed from each other in multiple rhythms. Moreover, the imagined SI and real SS showed similar activation patterns in α rhythms but differed in γ bands.

Motivated by the EEG and fMRI studies in tactile imagery, we picked ERD/ERS as the standard metrics to measure the dynamic variation of brain signals. It has been shown that brain function is associated with its frequency-specific brain activities. For instance, α rhythm in the frequency range of 8 to 13 Hz is not only modulated by attention and mental tasks but also sensitive to SS and movements [46]. Mu rhythm [8 13] Hz is another type of α rhythm that presents in the sensorimotor functions covering part of temporal, parietal, and central areas of the head. γ rhythm found in the similar region also characteristically appears with motor activities and sensory processing [47].

In attempt to better understand cortex activations, scalp signals were projected into the source domain. ESI as a technique to recover cortex signals was applied. Consistent with the research mentioned above, it was clearly observed that the task-induced ERD/ERS of the source domain responded in all 3 tasks within α rhythms (Fig. 2). The alpha activations that we observed within SI and SS groups covered across postcentral gyrus and precentral gyrus of central sulcus, fractional supramarginal gyrus, and superior parietal lobe but were widespread across the cortex in MI group. The fMRI study [48,49] states that primary motor (M1) and primary sensory (S1) cortices that are also defined as precentral gyrus and postcentral gyrus inside the central sulcus are strongly activated compared with other cortices during MI. Postcentral gyrus and precentral gyrus along with some superior and inferior parietals are activated during SI and SS, which was in line with the finding from fMRI studies [19,50]. Besides, frontal areas were activated in both α and γ rhythms especially within MI and SS groups. It was also worth noting that contralaterality of ERD/ERS distribution in α1 band was not obvious as compared to ERD/ERSs in α2. MI group showed bilateral activations especially in α1, while SI and SS revealed a more distinctive contralateral ERD activation.

Before conducting statistical analysis, all dipoles were subdivided into multiple independent neighbors called ROIs or scouts, and the number of ROIs in this study was 500 that were selected from 500 to 1,500 based on distances between each dipole and the cost of calculation. According to all results of statistical analysis, the number of ROIs (from 500 to 1,500) did not make significant influence on statistical differences between SI and MI or SI and SS. As observed in Fig. 2, differences between SI and MI for α bands (Figs. 3 and 4) were found within central sulcus such as paracentral, postcentral, and precentral gyri that were all related to the motor or sensorimotor area, and vice versa for RH case. Parietal lobe (mainly inferior, superior parietal, and supramarginal) and frontal lobe (mainly rostral middle and superior frontal) also showed distinctive differences for both hand cases between SI and MI in the α band. Central and parietal areas are associated with sensory and voluntary motor function according to the literature [51]. The differences around the somatosensory cortex between SI and MI demonstrate that SI and MI may have different modulation modes, one associating with tactile sensation but the other one dominant for motor control. Besides, statistical significances between SI and MI mainly occurred in alpha bands that are related to tactile sensation and body movement as mentioned above. Part of the parietal area is also responsible for somatosensory association, whereas frontal region is usually related to higher mental, motor control, and memory functions [52]. Those mentioned phenomena may further explain that different modulation modes between them indeed exist. Furthermore, analysis around sensorimotor areas for RH and LH revealed obvious task-related hemispheres, e.g., significant areas of LH mainly concentrating on the left hemisphere, while RH mainly over the right side.

Another interesting phenomenon was that temporal lobe also revealed statistical significances between MI and SI in α band. In concrete, for α1 in both hand cases, temporal lobe (mainly inferior, middle, superior temporal and fusiform) in bilateral hemispheres showed statistical differences between MI and SI according to the Desikan-Killiany atlas. In α2, temporal lobe mostly in the right hemisphere revealed statistical differences within MI versus SI group for both hands. The temporal lobe is usually involved in working and long-term memory [53]. Since MI and SI refer to imaging motor movement and sensation respectively, they may be correlated to 2 different working memory patterns which could explain why significance occurred in MI versus SI group.

Comparison between SI and SS for both hand cases has already been investigated in fMRI study [19] in which differences consistent with the finding in this paper were observed in 2 gamma bands (Fig. 5). Differences between SI and SS in γ1 for RH mostly occurred in right central sulcus and lateral occipital areas. SS is a passive process, while SI requires subjects to imagine the mental sensation and maintain their attention, so it is reasonable to infer that SI and SS do have some differences. However, it is interesting to note that differences between SI and SS in another EEG study [18] reveal an opposite conclusion where SI and SS differ from each other mainly in left central areas when right hands are stimulated and the statistical difference appears in mu and beta rhythms, not gamma in that study. Yakovlev et al. [18] utilized a 48-channel head cap, whereas a 128-channel high-density head cap was used in this study. Other studies [53–55] have revealed that high-density EEG signals have more potential to reconstruct high-frequency oscillations, which may explain why differences in SI versus SS group occurred within low-frequency bands in reference [18] but within gamma bands in this study. In LH case for γ2 rhythm, only caudal middle frontal, lateral occipital, and fusiform areas in the left hemisphere showed statistical differences. All subjects were right-handed; hence, it would be deduced that the result of SI versus SS was more distinctive in RH case.

Statistical results in the source domain can provide concrete information what scalp data cannot do about which neurophysiological source is significant among those groups. Significant areas found in those areas mentioned above just verified our speculation that MI and SI come from different brain regions especially within sensorimotor areas, while SI and SS do not reveal any sensorimotor difference in low-frequency bands.

One limitation of our study was that the common MRI template was used instead of subject-specific MRI scanning data. The subject-specific MRI can provide a more precise head model and thus get more detailed results of source oscillatory dynamics. Another limitation may be the method of ESI. There are plenty of ESI methods such as eLORETA, beamformer, and minimum norm estimation (MNE). There are plenty of ESI methods such as eLORETA, beamformer, and minimum norm estimation to further validate whether SI versus MI/SS group will illustrate similar statistical results by various imaging techniques is a new research topic that is not consistent with the main theme of this study. Moreover, there are researches [56,57] demonstrating that different imaging techniques have no statistically significant differences in source localization and do not influence statistical results. Therefore, permutation test with different inverse methods will be applied in future work, and a conventional source imaging technique named sLORETA was utilized for its excellent source localization and noise suppression capabilities. Future work should also collect individual fMRI data to get more precise head models, thereby more accurate source locations concerning different tasks. Besides, on the basis of the conclusion of this study and other applications combining SI, MI, and SS, the hybrid BCI, especially the online one, may associate stroke people in rehabilitation and reconstruct their motor and sensory circuits. The hybrid BCI also has the potential to assist myoelectric hand control through tactile and motor interaction [58] or solve the BCI-illiteracy problem by building a large multiclass BCI dataset with those 2 modalities (SI and MI).

## Data Availability

The data and code that support the findings of this study are available from the corresponding author upon reasonable request.
