# Supplementary material for: Neural Correlates of Motor/Tactile Imagery and Tactile Sensation in a BCI paradigm: A High-Density EEG Source Imaging Study
Source: Cyborg Bionic Syst. 2024 Jun 21;5:0118. doi: 10.34133/cbsystems.0118 (PMC11192147; doi:10.34133/cbsystems.0118)
Supplement: Supplementary 1 — Figs. S1 to S3 Table S1 [file cbsystems.0118.f1.pdf]

Supplementary Materials

The regions of interest were selected according to Figure S1 illustrated the image of Desikan-Killiany atlas, whereas Table S1 listed labels and hemsiphere corresponding to Figure S1.

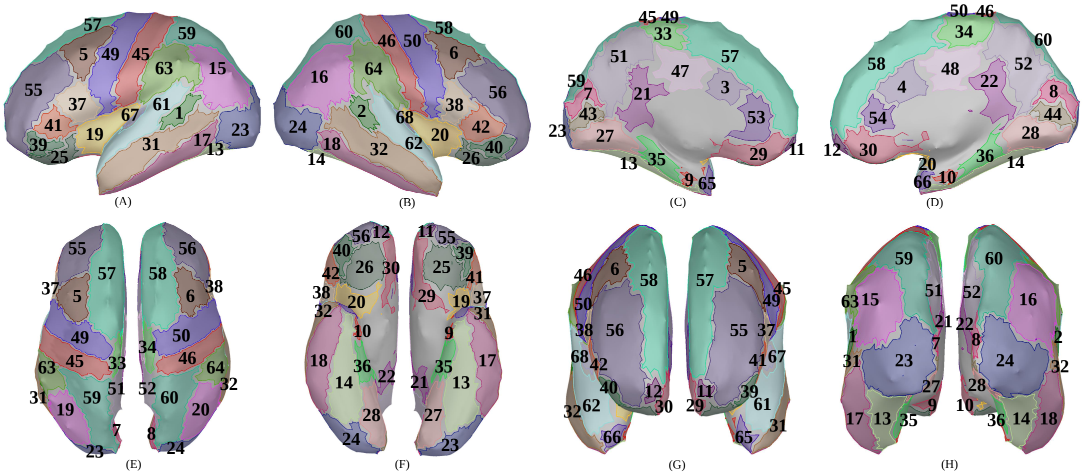

Figure S1: Regions of interest (ROIs), also named scouts, segmented according to the Desikan-Killiany atlas. (A) to (H) are brain maps of scout segmentations in the view of extern left, extern right, intern left, intern right, top, bottom, front and back in turn. Note: all scouts are labeled with numbers which are also listed in Table S1 in the form of names.

Figure S2 revealed the ERD/ERS distribution of beta rhythms [13 26]Hz. Although those three tasks illustrated obvious ERD/ERS activations, MI and SI did not showed any statistical differences within beta bands. In the left hand task, both $\beta1$ and $\beta2$ revealed distinctive contralateral activations (ERD) and vice versa for RH.

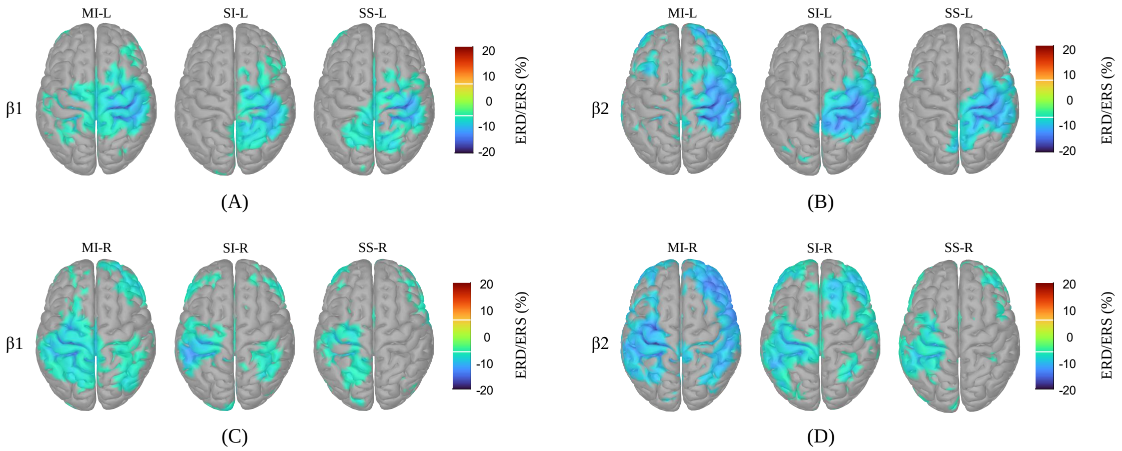

Figure S2: Brain map of grand-averaged ERD/ERS within β bands. (A) are LH ERD/ERS activation in $\beta1$. (B) are ERD/ERS LH activation in $\beta2$. (C) are RH ERD/ERS activation in $\beta1$. (D) are RH ERD/ERS activation in $\beta2$.

Table S1: Tabel of Desikan-Killiany altas

Number	Label	Hem	Number	Label	Hem
1	bankssts	R	35	parahippocampal	R
2	bankssts	L	36	parahippocampal	L
3	caudal anterior cingulate	R	37	parsopercularis	R
4	caudal anterior cingulate	L	38	parsopercularis	L
5	caudal middle frontal	R	39	parsorbitalis	R
6	caudal middle frontal	L	40	parsorbitalis	L
7	cuneus	R	41	parstriangularis	R
8	cuneus	L	42	parstriangularis	L
9	entorhinal	R	43	pericalcarine	R
10	entorhinal	L	44	pericalcarine	L
11	frontal pole	R	45	postcentral	R
12	frontal pole	L	46	postcentral	L
13	fusiform	R	47	posterior cingulate	R
14	fusiform	L	48	posterior cingulate	L
15	inferior parietal	R	49	precentral	R
16	inferior parietal	L	50	precentral	L
17	inferior temporal	R	51	precuneus	R
18	inferior temporal	L	52	precuneus	L
19	insula	R	53	rostral anterior cingulate	R
20	insula	L	54	rostral anterior cingulate	L
21	isthmus cingulate	R	55	rostral middle frontal	R
22	isthmus cingulate	L	56	rostral middle frontal	L
23	lateral occipital	R	57	superior frontal	R
24	lateral occipital	L	58	superior frontal	L
25	lateral orbitofrontal	R	59	superior parietal	R
26	lateral orbitofrontal	L	60	superior parietal	L
27	lingual	R	61	superior temporal	R
28	lingual	L	62	superior temporal	L
29	medial orbitofrontal	R	63	supramarginal	R
30	medial orbitofrontal	L	64	supramarginal	L
31	middle temporal	R	65	temporal pole	R
32	middle temporal	L	66	temporal pole	L
33	paracentral	R	67	transverse temporal	R
34	paracentral	L	68	transverse temporal	L

Moreover, signals were filtered from 8 to 20 Hz before feature extraction. A Common Spatial Pattern (CSP) over the sensorimotor cortex was used to reduce the number of scouts and extract features of the source signal [59]. Linear discriminative analysis (LDA) was utilized for the classification. A 10-fold cross-validation was performed repeatedly in each block per subject. The classification accuracy of three blocks per subject in source space was listed in Figure S3. Among those three tasks, subject 8 had the highest classification accuracy (82.58±0.82%) in MI task while the highest accuracies in SI and SS were 91.22±0.65% and 94.41±0.55% of subject 4 respectively. The accuracy of SS task without doubt gained the highest average accuracy among those three tasks with 74.05±10.81% across all subjects.

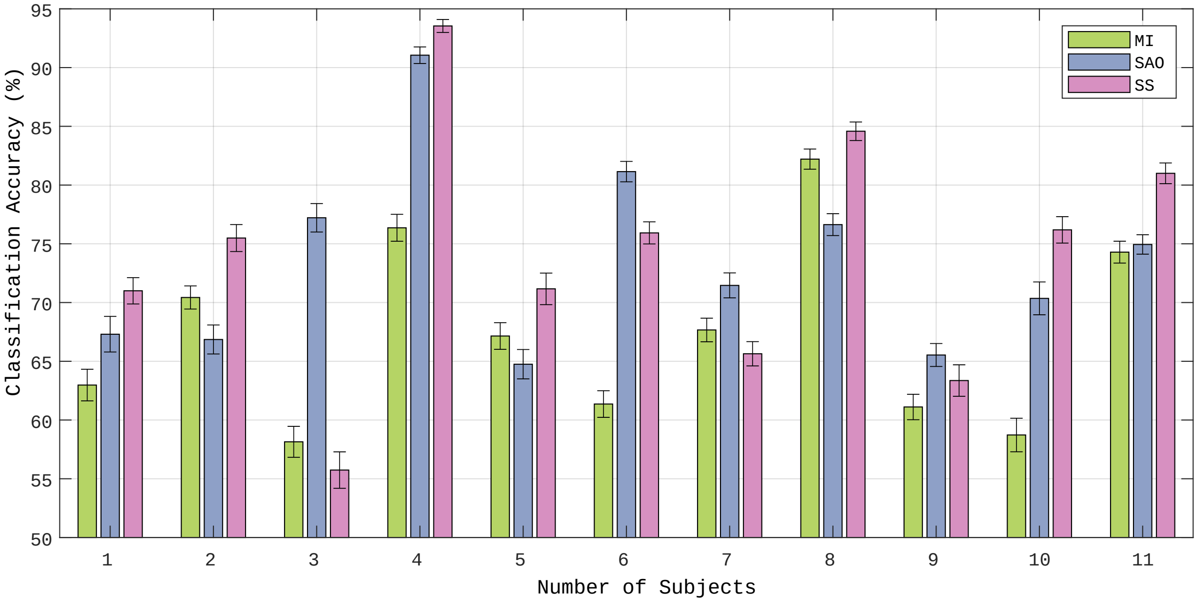

Figure S3: Classification accuracy of MI/SI/SS for 11 subjects.
